# Separation and Purification of *Astragalus membranaceus* Polysaccharides by Deep Eutectic Solvents-Based Aqueous Two-Phase System

**DOI:** 10.3390/molecules27165288

**Published:** 2022-08-19

**Authors:** Bangfu Liu, Zhijian Tan

**Affiliations:** 1 Hunan Electronic Information Industry Institute, Changsha 410012, China; 2 Institute of Bast Fiber Crops & Center of Southern Economic Crops, Chinese Academy of Agricultural Sciences, Changsha 410205, China

**Keywords:** *Astragalus membranaceus* polysaccharides, deep eutectic solvents, solid–liquid extraction, aqueous two-phase system, separation and purification

## Abstract

(1) Background: Aqueous two-phase systems (ATPSs) have been widely used in the separation and purification of bioactive substances in recent years. (2) Methods: In this study, deep eutectic solvents (DESs)-based ATPSs were employed for the extraction and separation of *Astragalus membranaceus* polysaccharides (AMP). The optimal DES (choline chloride:urea = 1:1) was first screened to extract AMP, and the effect of DES concentration, solid–liquid ratio, pH, extraction temperature, and extraction time on the extraction yield of AMP were investigated. (3) Results: The maximum extraction yield was 141.11 mg/g under the optimum conditions. AMP was then preliminarily purified by ATPS, to further realize the recycling and reuse of DES. The effect of type of salts, salt concentration, and extraction temperature on extraction efficiency was investigated. The extraction efficiency was 97.85% for AMP under the optimum ATPS conditions. Finally, the obtained AMP was studied by molecular weight determination, infrared spectroscopy analysis, and monosaccharide composition analysis. (4) Conclusions: This ATPS extraction based on DESs is simple, environmentally friendly, low-cost, and has high extraction efficiency, which provides new ideas for the extraction of plant polysaccharides and other bioactive compounds.

## 1. Introduction

*Astragalus membranaceus*, a genus of leguminosae in dicotyledons, is a kind of traditional Chinese medicinal plant and an important raw material of health food [1]. Studies have shown that the bioactive components of *Astragalus membranaceus* include polysaccharides, saponins, flavonoids, amino acids, and other compounds [2]. *Astragalus membranaceus* polysaccharide (AMP) is an important active component that has the functions of bacteriostasis, anti-inflammatory, enhancing immunity, and anti-aging [3].

So far, the main methods of AMP extraction include aqueous extraction, enzyme-assisted extraction, ultrasonic extraction, and alkali extraction [4,5,6,7,8]. Aqueous extraction has the disadvantages of low extraction efficiency and is very time-consuming. Although the enzyme-assisted extraction method has high extraction efficiency, it has a high requirement for environmental conditions, so it is not suitable for industrial production. Ultrasonic extraction has high extraction efficiency, but the strong mechanical vibration of the ultrasonic procedure may also destroy the structure of the polysaccharides and reduce the activity of the polysaccharides [6]. The alkali extraction not only has low extraction efficiency but also reduces the bioactivity of polysaccharides.

In 2003, deep eutectic solvents (DESs) were first prepared by Abbott and his coworkers, using choline chloride as a hydrogen bond acceptor (HBA) and urea as hydrogen bond donor (HBD) [9]. In 2019, Coutihno et al. improved the definition of DES, which provided a solid theoretical basis for further research and application of DES [10]. In the past, traditional organic solvents were widely used to extract bioactive substances. However, these organic solvents are volatile and toxic, which can cause environmental pollution [11]. With the increasing requirements of the public for “green” and “environmental protection”, green chemistry has become one of the research hotspots in recent years. As environmentally friendly solvents, most DESs have the advantages of low toxicity, easy preparation, and good stability [12,13]. Therefore, DESs can be used as good substitutes for traditional organic solvents. At present, DESs have been widely used in the extraction of various natural substances, such as alkaloids [14], polyphenols [15,16], flavonoids [17], hemicellulose [18], lignin [19], etc. DESs are widely used in the extraction of bioactive compounds, but it is difficult to separate DESs from the target products after extraction. Therefore, switchable DESs have been developed, but they still have the disadvantages of difficult preparation and high cost [20].

An aqueous two-phase system (ATPS) is a mixed aqueous solution of either two hydrophilic polymers or a hydrophilic polymer and a salt, which will form two insoluble phases at appropriate concentrations [21,22]. When the target compounds are added to ATPS, they are separated into different phases due to the influence of surface properties and various interaction forces (such as hydrogen bond interaction and ionic bond interaction) [23,24]. Compared with the traditional oil–water solvent extraction systems, ATPS extraction has the advantages of mild operation conditions, excellent biocompatibility, and simple operation [25,26]. At present, ATPSs have been widely used in the extraction and separation of proteins [27,28], antibiotics [29], flavonoids [30], polysaccharides [31,32], and so on. DESs-based ATPSs have drawn more attention in the last few years, which were developed for the extraction of proteins [33], anthraquinones [34], DNA [35], 5-hydroxymethylfurfural (5-HMF) [36], and so on.

In this study, AMP was extracted by using DESs as the extractants to obtain the crude extract; the effect of DES concentration, solid–liquid ratio, pH, extraction temperature, and extraction time on the extraction yield of AMP was studied. Then, ATPS was constructed for the preliminary purification of AMP, and the effect of the type of salt, salt concentration, and extraction temperature on the extraction efficiency was investigated. Finally, AMP was separated from DES by recycling and reusing the DES.

## 2. Results and Discussion

### 2.1. Selection of the Optimal DES

**Figure 1:**
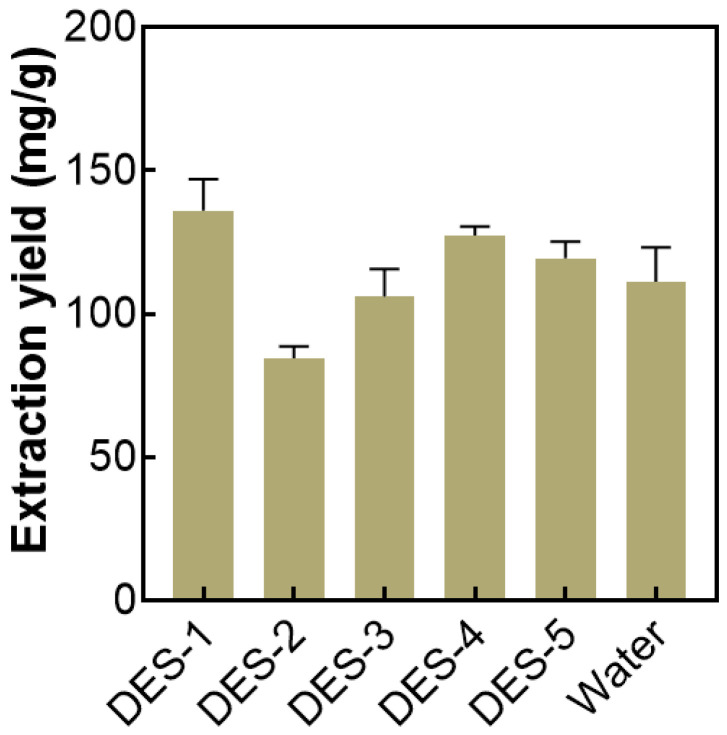
Effect of different DESs on AMP extraction. The extraction conditions were as follows: DESs concentration was 80 wt%, liquid–solid ratio was 30:1, pH was not adjusted, the extraction temperature was 60 °C, and the extraction time was 60 min.

Five DESs were selected as the extractants to extract AMP, and the extraction of AMP using water was used as a control. As shown in Figure 1, the extraction yield of AMP extracted with DES-1 is much higher than that of other DESs and water. This can be attributed to the fact that the HBD in DES-1 is alkaline urea, which is conducive to polysaccharide extraction. It is reported that the polarity, viscosity, and other properties of DESs may also affect the extraction efficiency of DESs; moreover, the hydrogen bond interaction, hydrophobic interaction, van der Waals force, and other interactions between DESs and the target materials can also affect the extraction ability of DESs [37,38]. Therefore, DES-1 was selected for the following studies.

### 2.2. Single-Factor Experiments of Extraction AMP

In this study, AMP was extracted by solid–liquid extraction. To improve the extraction yield, several main influencing factors (including DES concentration, solid–liquid ratio, pH, extraction temperature, and extraction time) were studied in detail.

**Figure 2:**
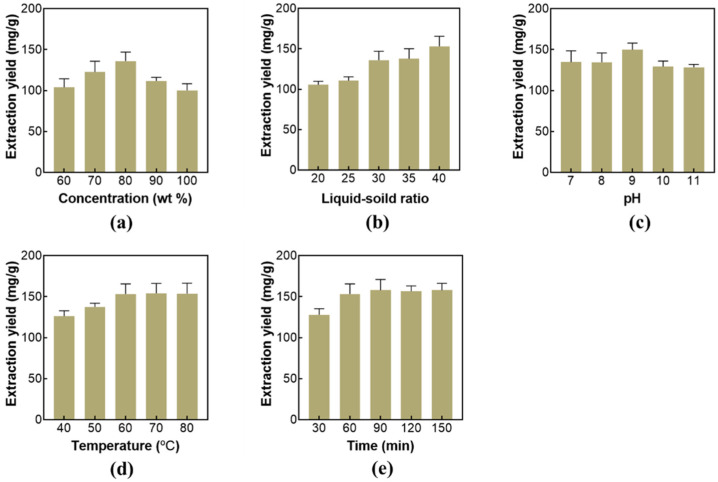
The single-factor experiments of AMP extraction. (**a**) Effect of DESs concentration on AMP extraction. The extraction conditions were as follows: liquid–solid ratio was 30:1, pH was not adjusted, the extraction temperature was 60 °C, and the extraction time was 60 min. (**b**) Effect of the liquid–solid ratio on AMP extraction. The extraction conditions were as follows: DESs concentration was 80 wt%, pH was not adjusted, the extraction temperature was 60 °C, and the extraction time was 60 min. (**c**) Effect of pH on AMP extraction. The extraction conditions were as follows: DESs concentration was 80 wt%, the liquid–solid ratio was 40:1, the extraction temperature was 60 °C, and the extraction time was 60 min. (**d**) Effect of the extraction temperature on AMP extraction. The extraction conditions were as follows: DESs concentration was 80 wt%, the liquid–solid ratio was 40:1, pH was not adjusted, and the extraction time was 60 min. (**e**) Effect of the extraction time on AMP extraction. The extraction conditions were as follows: DES concentration was 80 wt%, the liquid–solid ratio was 40:1, pH was not adjusted, and the extraction temperature was 60 °C.

DES-1 was selected as the optimal extractant for AMP extraction. The effect of DESs concentration from 60 to 90 wt% on the extraction yield of AMP was studied. The results are shown in Figure 2a, and it can be seen that the extraction yield first increases and then decreases with the increase in DES concentration. When the concentration of DES is 80 wt%, the extraction yield reaches 135.92 mg/g. Because the hydrogen bonding forces are weak when the DESs concentration is small. However, the high DESs concentration will increase the solution viscosity, which is also adverse for extraction. Therefore, 80 wt% DESs concentration was selected for further studies.

The effect of the liquid–solid ratio from 20:1 to 40:1 on the extraction yield of AMP was studied in this work. As shown in Figure 2b, the extraction yield continues to increase with the increase in the liquid–solid ratio. When the liquid–solid ratio is 40:1, the extraction yield reaches 153.07 mg/g. With the increase in the solvent, AMP can dissolve more in the solvent, while excessive solvent will cause waste. The results are similar to the reported literature [39]. Therefore, a liquid–solid ratio of 40:1 was selected for the extraction.

The extraction pH from 7 to 11 was studied. The pH of the system was adjusted by a phosphate buffer solution. It can be seen from Figure 2c that the extraction yield did not change significantly with pH changing. Since the pH of the system itself is 9.2, so the system pH was not adjusted in the following studies.

Extraction temperatures from 40 to 80 °C were investigated. As shown in Figure 2d, the extraction yield increases significantly with the increase in temperature from 40 to 60 °C. When the extraction temperature is 60 °C, the extraction yield reaches 153.07 mg/g. The increase in temperature can reduce the viscosity of DESs and accelerate the mass transfer efficiency of polysaccharides, so the extraction yield increases. However, when the temperature is higher than 60 °C, the extraction yield has no obvious change. Therefore, 60 °C was selected as the optimal temperature.

The effects of extraction times from 30 to 150 min were studied. As shown in Figure 2e, when the extraction time is 30–90 min, the extraction yield of AMP increases significantly with the increase in extraction time. When the extraction time was 30 min, the extraction yield was 127.93 mg/g, and when the extraction time was 90 min, the extraction yield reached 157.78 mg/g. The extraction yield changes little with the further increase in extraction time. The longer extraction time will enhance the cost. Therefore, the extraction time of 90 min was selected.

### 2.3. Extraction and Preliminary Purification of AMP by ATPS

The types of salt, salt concentration, and temperature are all regarded as critical factors influencing ATPS extraction, so it is necessary to investigate these factors.

#### 2.3.1. Effect of Salt Type on ATPS Extraction

Two representative salts (K_2_HPO_4_ and K_3_PO_4_) were selected as salting-out reagents. As shown in the results, the AMP extraction efficiency of the DES/K_3_PO_4_ system (94.59%) is much higher than that of the DES/K_2_HPO_4_ system (56.44%). This can be attributed to the stronger alkalinity of K_3_PO_4_, which is conducive to polysaccharides extraction. Thus, the DES/K_3_PO_4_ system was chosen for further studies.

#### 2.3.2. Effect of Salt Concentration on ATPS Extraction

**Figure 3:**
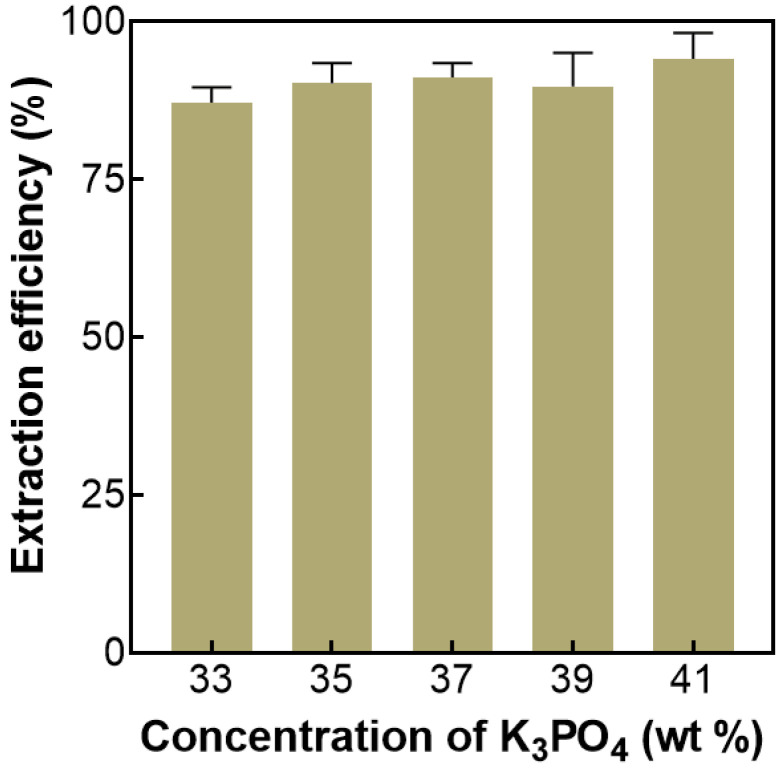
Effect of K_3_PO_4_ concentration on the extraction efficiency of AMP. The fixed extraction condition: the extraction temperature was 15 °C.

The salt concentration is a very important factor in salting-out extraction, so it is necessary to study the effect of salt concentration on ATPS extraction. The effect of salt concentrations in the range of 33–41 wt% was investigated in this work. The results are shown in Figure 3, and it can be seen from the results that the extraction efficiency of AMP (94.14%) is the highest when the salt concentration is 41 wt%. The increase in salt concentration can improve the salting-out ability and facilitate phase separation. Since the salt solution was saturated, the salt concentration could not be increased further. Therefore, a salt concentration of 41 wt% was selected for subsequent studies.

#### 2.3.3. Effect of Temperature on ATPS Extraction

**Figure 4:**
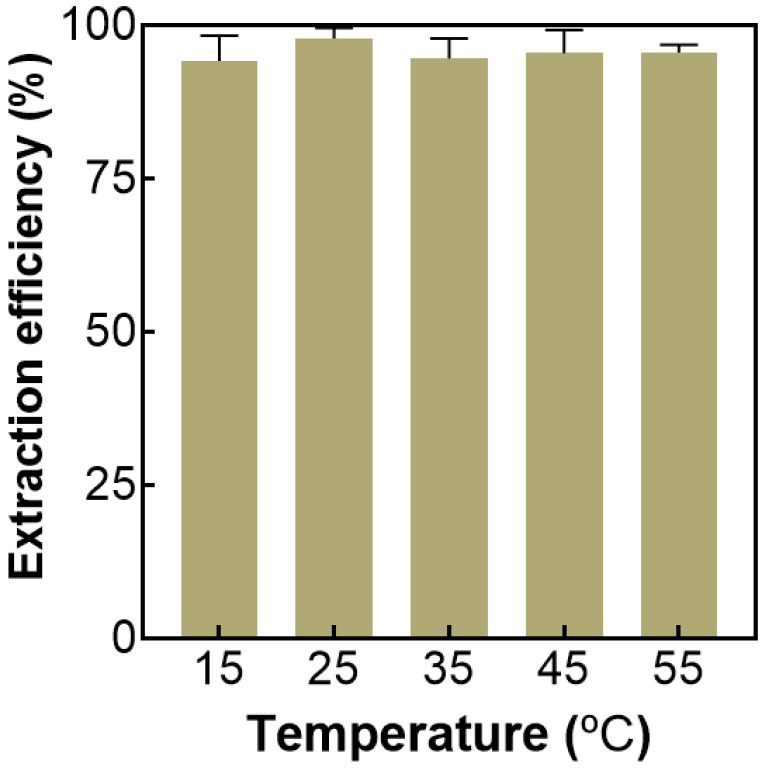
Effect of extraction temperature on the extraction efficiency of AMP. The fixed extraction condition: the K_3_PO_4_ concentration was 41 wt%.

The effect of temperature from 15 to 55 °C on ATPS extraction was investigated. The results in Figure 4 show that temperature has no significant effect on the extraction efficiency of AMP. Therefore, to facilitate the experiment, the follow-up experiments were conducted at room temperature.

### 2.4. Recycling Studies

**Figure 5:**
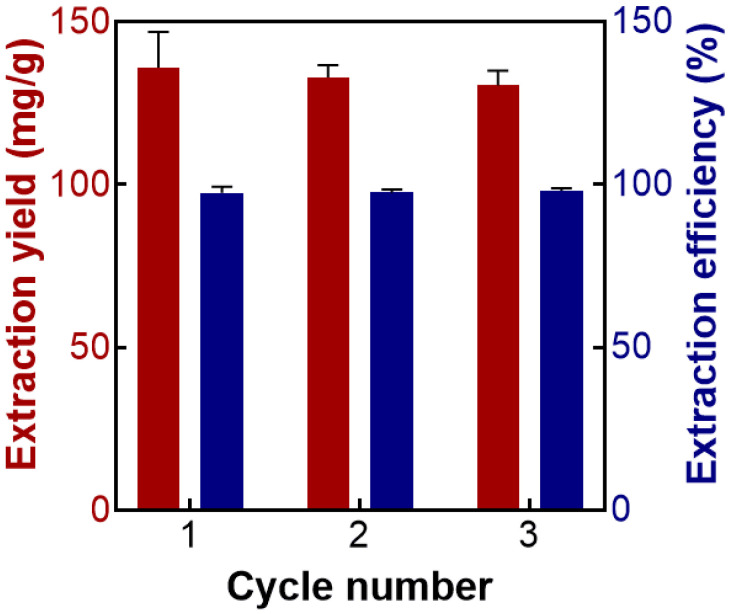
The extraction yield (*Y*, mg/g) and extraction efficiency (%) of AMP in the recycling tests.

The DES-rich phase was separated, the water in DES was removed by drying, and the AMP was extracted according to the optimal conditions. According to this procedure, the DES was recycled and reused for three cycles. As shown in Figure 5, the extraction yield and extraction efficiency of AMP decrease slightly after three cycles. The extraction yield and extraction efficiency of AMP in the third cycle are 131.65 mg/g and 97.51%, respectively. These results proved that DES has good recycling performance in the ATPS extraction process.

### 2.5. The Analysis of AMP

**Figure 6:**
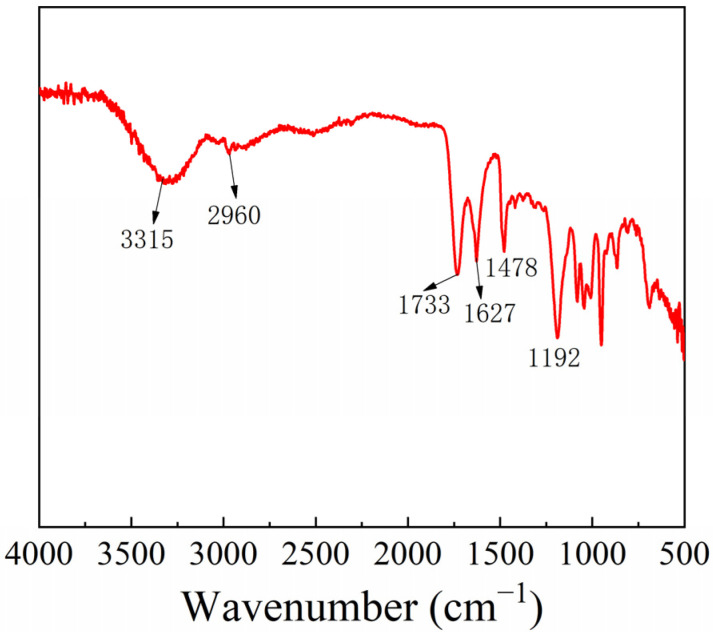
The FT-IR spectra of AMP.

The molecular weight, monosaccharide composition, and FT-IR for AMP were analyzed. The structural characteristics of polysaccharides, including glycosidic bonds and functional groups, can be analyzed by FT-IR spectroscopy. The FT-IR is shown in Figure 6, which confirms the typical characteristic bands of AMP. The strong peak at 3315 cm^−1^ is ascribed to the stretching vibration of O-H. The weak peak at 2960 cm^−1^ is related to the stretching vibration of C-H. The absorption bands at 1733 and 1627 cm^−1^ are caused by C=O asymmetric and symmetric stretching vibrations. The bond at 1478 cm^−1^ is the symmetrical deformation vibration of C-H, and the absorption around 1192 cm^−1^ is the stretching vibrations of the C-O-C and glycosidic bond [40]. The molecular weight of AMP is 4.86 kDa (Table S1 and Figure S1). The monosaccharide analysis of AMP is shown in Table 1 and Figure S2; it can be seen from the results that glucose, rhamnose, and fucose are the main monosaccharides of AMP.

## 3. Materials and Methods

### 3.1. Materials and Reagents

Dried *Astragalus membranaceus* was purchased from a local drugstore in Changsha, Hunan Province. The dried *Astragalus* was crushed, sieved (60 mesh), and then stored on a dry and cool site. Choline chloride, urea, ethylene glycol, glycerin, oxalic acid, lactic acid, phenol, sulfuric acid, potassium phosphate, and dipotassium phosphate were all purchased from Aladdin Reagent Co., Ltd. (Shanghai, China). All of the chemicals used in this study were of analytical grade.

### 3.2. Preparation and Characterization of DESs

The DESs were prepared by the heating method [12]. The HBDs and HBAs were stirred and heated at a certain molar ratio at 80 °C for 2 h to obtain a clear liquid. The details of these DESs are shown in Table 2. The characterizations of DESs (HBA: choline chloride) are shown in Figures S3–S7.

### 3.3. Extraction of Polysaccharides Using DESs

In a 10 mL centrifuge tube, 0.2 g of dried *Astragalus* powder and a certain volume of DESs were added. AMP was extracted by ultrasonic-assisted heating. After centrifugation, the supernatant was withdrawn to determine the polysaccharide content. The content of polysaccharides was determined by the phenol–sulfuric acid method. The standard curve was obtained using glucose concentration as the abscissa and the absorbance at the 490 nm wavelength as the ordinate. The standard curve is shown in Equation (1). The standard glucose solution is in the concentration range of 2.5–12.5 μg/mL.

(1) $$y=32.616x-0.0104\;\left(R^{2}=0.9998\right)$$

The extraction yield (*Y*, mg/g) of AMP is calculated by Equation (2).

(2) $$Y\;\left(\mathrm{mg}/g\right)=\frac{\mathrm{Mass}\;\mathrm{of}\;\mathrm{determined}\;\mathrm{AMP}\;\left(\mathrm{mg}\right)}{\mathrm{Mass}\;\mathrm{of}\;\mathrm{dried}\;Astragalus\;membranaceus\;\mathrm{powder}\;\left(g\right)}\;$$

### 3.4. Separation of Polysaccharides by ATPS

In a centrifuge tube, the crude polysaccharides extract and a certain amount of salt solution (K_3_PO_4_ or K_2_HPO_4_) were added, and the mixture was fully stirred. After centrifugation, two phases were formed. The volume of each phase was recorded, and the content of polysaccharides in the top and bottom phases were determined, respectively. The extraction efficiency (E, %) of AMP in the bottom phase was calculated by Equation (3).

(3) $$E\left(\%\right)=\frac{C_{1}\times V_{1}}{C_{2}\times V_{2}+C_{1}\times V_{1}}\times 100\%\;$$

where *C*_1_ and *V*_1_ represent the AMP concentration and volume of the bottom phase, respectively. *C*_2_ and *V*_2_ represent the AMP concentration and volume of the top phase, respectively.

### 3.5. Determination of the Molecular Weight

The molecular weight of AMP was determined by HPLC-RID (LC-20, Shimadzu, Japan; RID-20, Shimadzu, Japan) equipped with an aqueous gel column (TSKgel GMPWXL, 7.5 mm × 300 mm, TOSOH, Tokyo, Japan). The parameter settings were as follows: the injection volume was 20 µL; the mobile phase was 0.1 mol/L NaNO_3_ + 0.06% NaN_3_ solution; the flow rate was 0.6 mL/min; the column temperature was 35 °C.

### 3.6. Analysis of the Monosaccharide

The Ultimate-3000 HPLC equipped with the Xtimate C_18_ column (4.6 × 200 mm, 5 µm, Eka Nobel, Sweden) was used to analyze the monosaccharide constituents of the obtained AMP according to the reported method [41]. The parameter settings were as follows: a UV–Vis detector was used at the detection wavelength of 250 nm; the mobile phase was 0.05 mol/L potassium dihydrogen phosphate solution (pH = 6.7) and acetonitrile at a ratio of 83:17; the flow rate of 1.0 mL/min; the column temperature was 30 °C; the injection volume was 20 µL.

## 4. Conclusions

In this study, ATPS based on DESs has been developed for the extraction and preliminary purification of AMP. DES-1 (choline chloride:urea = 1:1) was selected as the optimal extractant. The extraction yield was 141.11 mg/g when the DES concentration was 80 wt%, the solid–liquid ratio was 1:40, the pH was not adjusted, the extraction temperature was 60 °C, and the extraction time was 90 min. Afterward, AMP was preliminarily purified by ATPS extraction. The extraction efficiency was 97.85% at the K_3_PO_4_ concentration of 41.0 wt% and extraction temperature of 25 °C. The extraction yield and extraction efficiency of AMP decreased slightly after three cycles, which proved that the DESs used in this study had good cyclic stability. The molecular weight determination, monosaccharide analysis, and FT-IR analysis of the obtained AMP were studied. This ATPS based on DESs can be effectively used for the extraction of AMP and allow for the recycling and reuse of extractants, which provides a new idea for the extraction of plant polysaccharides and other bioactive ingredients.

## Figures and Tables

**Table 1:** The monosaccharide composition of AMP.

Monosaccharide Composition	Percentage (%)
Mannose	0.3352
Ribose	0.079652
Rhamnose	7.044621
Glucuronic acid	4.45985
Galacturonic acid	0.744248
Glucose	76.11869
Galactose	4.757241
Xylose	0.076693
Arabinose	0.982739
Fucose	5.41545

**Table 2:** The details of the prepared DESs (HBA: choline chloride).

No.	HBDs	Molar Ratios
DES-1	Urea	1:1
DES-2	Glycol	1:1
DES-3	Glycerol	1:1
DES-4	Oxalate	1:1
DES-5	Lactic acid	1:1

## Supplementary Materials

The following are available online at https://www.mdpi.com/article/10.3390/molecules27165288/s1, Table S1: The molecular weight of AMP. Figure S1: High-performance gel permeation chromatograms for AMP. Figure S2: The HPLC chromatograms for monosaccharide analysis of AMP. Figure S3: The FT-IR spectra for DES-1. Figure S4: The FT-IR spectra for DES-2. Figure S5: The FT-IR spectra for DES-3. Figure S6: The FT-IR spectra for DES-4. Figure S7: The FT-IR spectra for DES-5.

## Sample Availability

Samples of the compounds *Astragalus membranaceus* are available from the authors.
