# Supplementary material for: Separation and Purification of Astragalus membranaceus Polysaccharides by Deep Eutectic Solvents-Based Aqueous Two-Phase System
Source: Molecules. 2022 Aug 19;27(16):5288. doi: 10.3390/molecules27165288 (PMC9412596; doi:10.3390/molecules27165288)
Supplement: Supplementary file 1 [file molecules-27-05288-s001.zip › molecules-1781756-supplementary.pdf]

Supplementary Materials

Separation and Purification of *Astragalus Membranaceus* Polysaccharides by Deep Eutectic Solvents-based Aqueous Two-Phase System

Bangfu Liu [1], Zhijian Tan [2,*]

[1] Hunan Electronic Information Industry Institute, Changsha 41001, China
[2] Institute of Bast Fiber Crops & Center of Southern Economic Crops, Chinese Academy of Agricultural Sciences, Changsha 410205, China
* Correspondence: tanzhijian@caas.cn; Tel.: +86-731-8899-8517

Table S1. The molecular weight of AMP

	Peak 1 (67.4%)	Peak 2 (33.6%)	Average
Mw (Da)	6654	371	4856

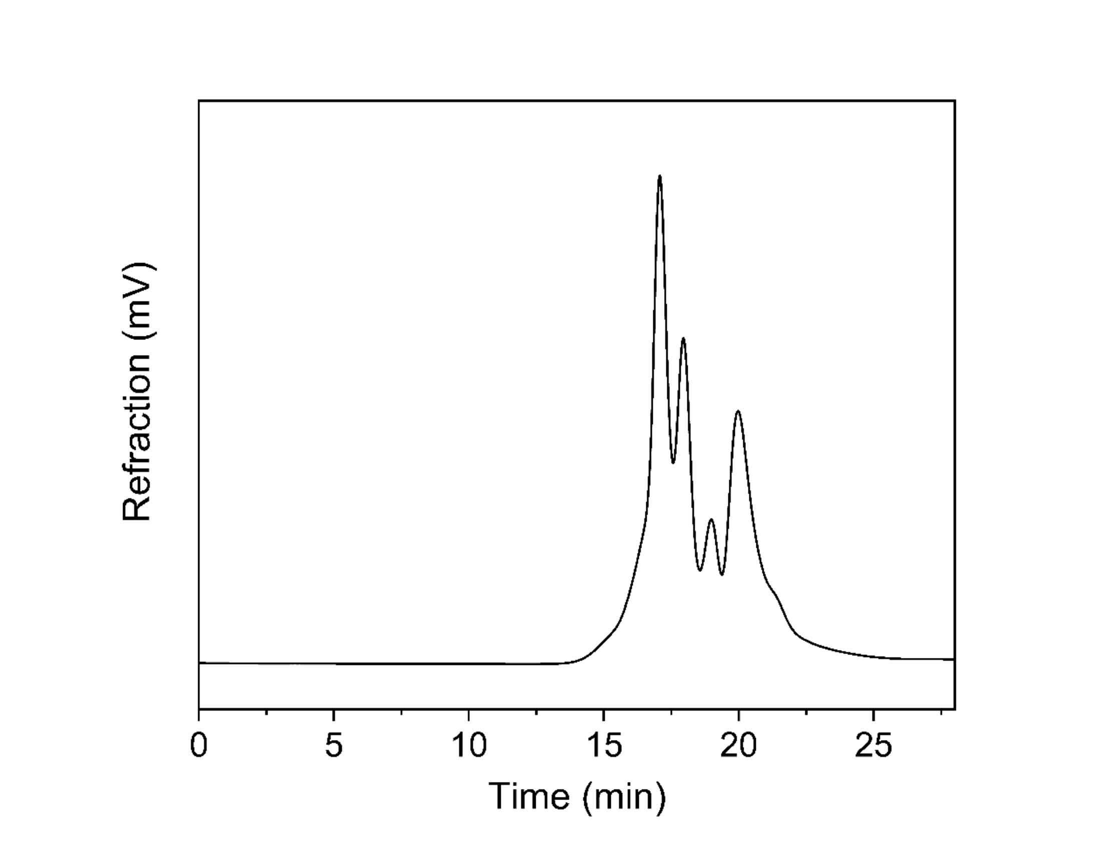

Figure S1. High-performance gel permeation chromatograms for AMP.

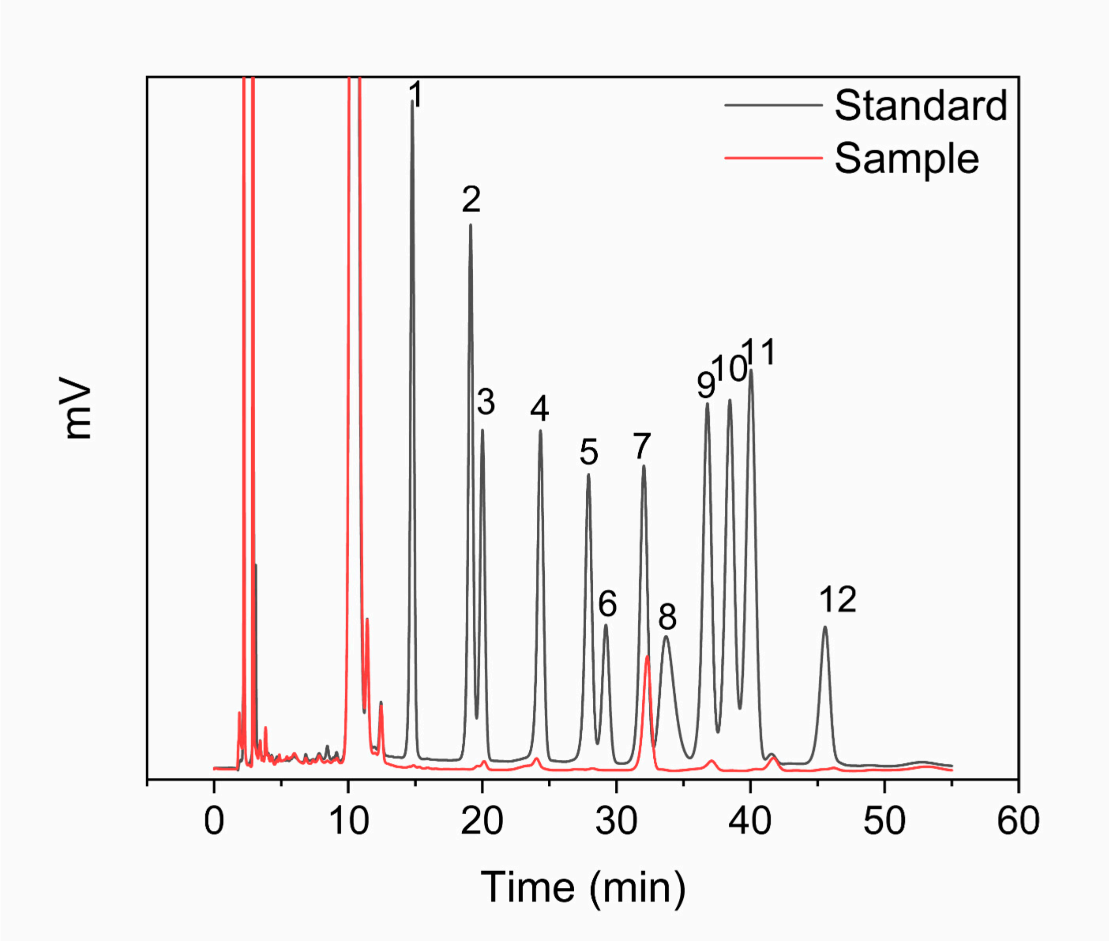

1- Mannose
2- Ribose
3- Rhamnose
4- Glucuronic acid
5- Galacturonic acid
6- N-acetyl-glucosamine
7- Glucose
8- N-acetyl-galactosamine
9- Galactose
10- Xylose
11- Arabinose
12- Fucose

Figure S2. The HPLC chromatograms for monosaccharide analysis of AMP

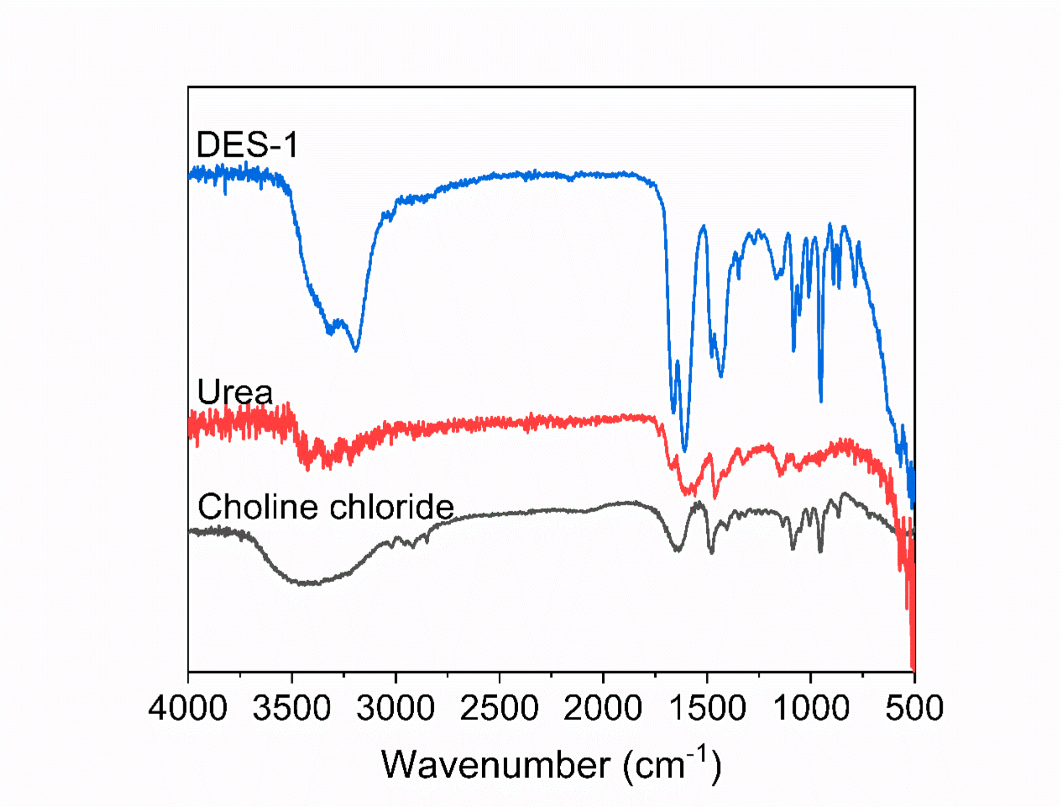

Figure S3. The FT-IR spectra for DES-1

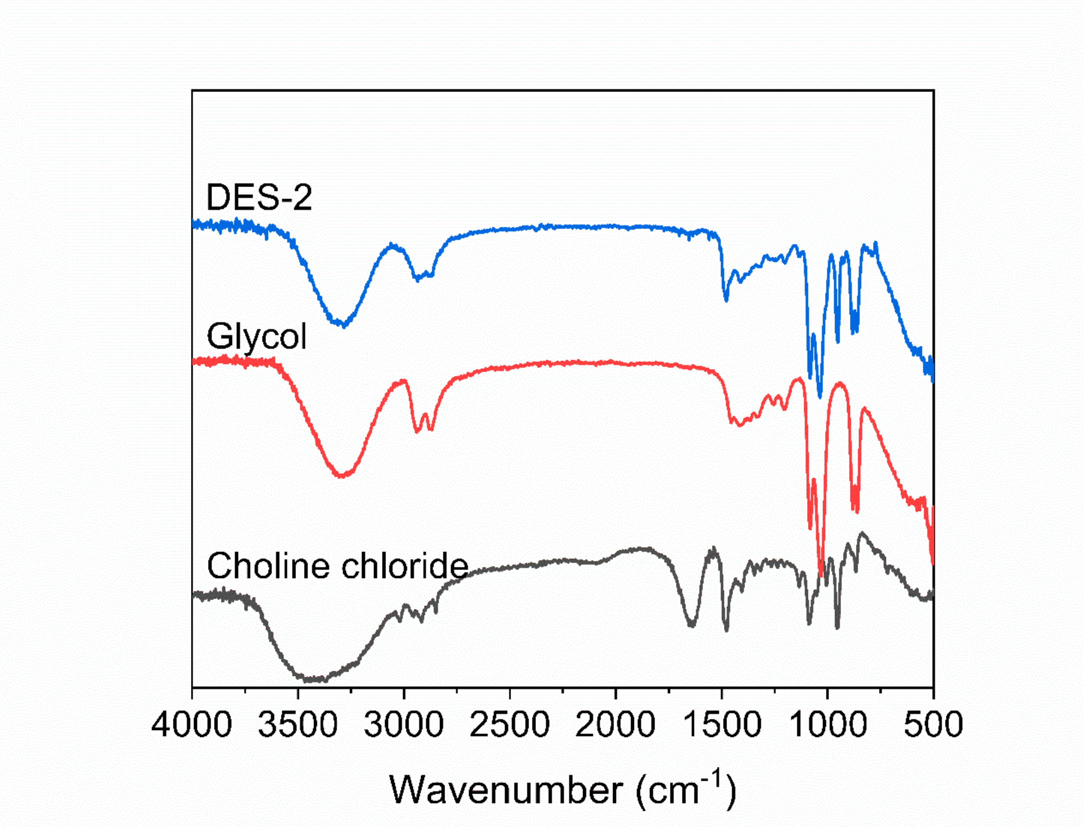

Figure S4. The FT-IR spectra for DES-2

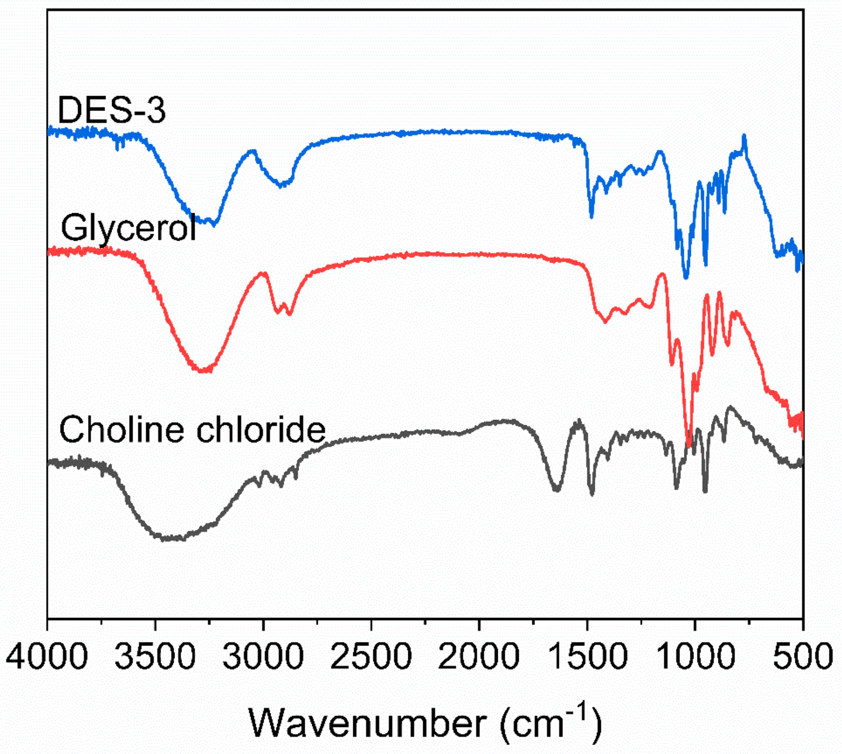

Figure S5. The FT-IR spectra for DES-3

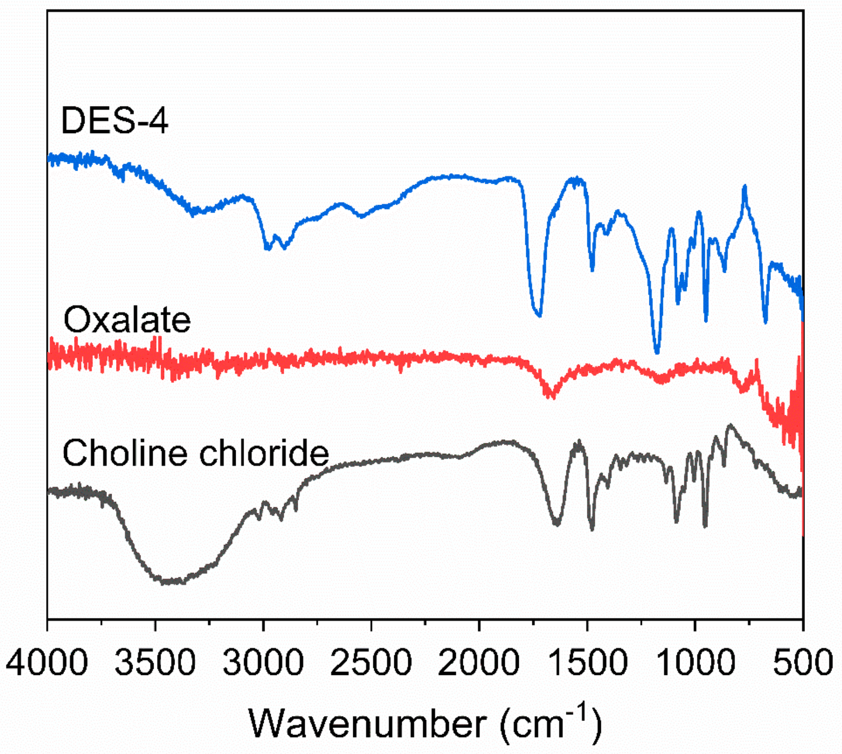

Figure S6. The FT-IR spectra for DES-4

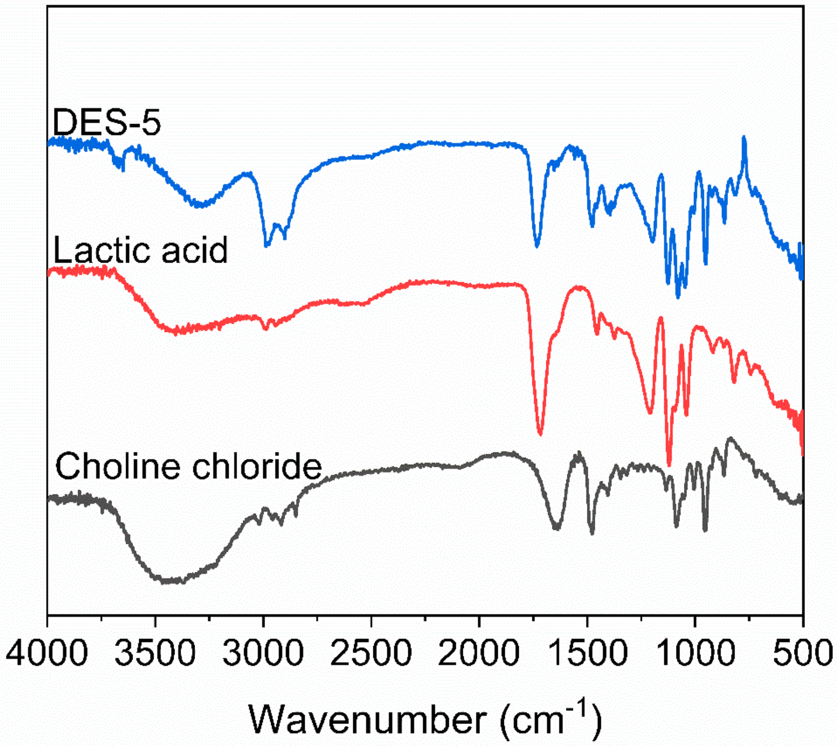

Figure S7. The FT-IR spectra for DES-5
